# EgSPE, a secreted protein from *Epichloë gansuensis*, modulates symbiotic establishment and host drought tolerance

**DOI:** 10.1186/s12870-026-09133-1

**Published:** 2026-05-30

**Authors:** Haijuan Zhang, Haotian Shi, Chunjie Li, Lei Lei

**Affiliations:** State Key Laboratory of Herbage Improvement and Grassland Agro- ecosystems, College of Pastoral Agriculture Science and Technology, Lanzhou University, Lanzhou, 730020 China

**Keywords:** *Epichloë* endophyte, Transformation system, Secreted protein, Drought tolerance

## Abstract

**Background:**

*Epichloë* endophytes form beneficial symbioses with cool-season grasses, enhancing host tolerance to abiotic stresses such as drought while maintaining normal plant growth. However, the molecular mechanisms underpinning this symbiosis, particularly the role of fungal-secreted protein, remain largely unexplored.

**Results:**

In this study, we identify EgSPE, a secreted protein from *Epichloë gansuensis*, as a key regulator of symbiotic establishment and host drought adaptation in drunken horse grass (*Achnatherum inebrians*). Transcriptome profiling during host colonization revealed *EgSPE* as a strongly induced gene encoding a secreted protein. Functional characterization facilitated by a substantially improved transformation system demonstrates that EgSPE is critical for fungal growth and efficient host colonization, as its deletion severely disrupted symbiotic establishment. Notably, EgSPE activates the host drought-responsive signaling by inducing the marker gene *RD29A* in a heterologous system (*Nicotiana benthamiana*) and upregulating stress-related genes (*AiRD22*, *AiNAC5*, and *AiABA1*) in its native host (*A. inebrians*). Consistently, only the *E. gansuensis* wild-type and OE-*EgSPE* strains enhanced host drought resistance, whereas the Δ*egspe* mutants failed to confer this benefit.

**Conclusions:**

In summary, our research findings identify EgSPE as a fungal protein that plays an important role in the establishment of symbiosis and in the host’s drought response, providing strong evidence for how *E. gansuensis* promotes abiotic stress tolerance in grasses.

**Supplementary Information:**

The online version contains supplementary material available at 10.1186/s12870-026-09133-1.

## Background

*Epichloë* endophytes establish mutualistic symbioses with more than one hundred cool-season grass species, primarily within the subfamily Pooideae (e.g., *Festuca*, *Lolium*, and *Poa* spp.) [1]. These symbionts confer enhanced tolerance against a broad spectrum of biotic and abiotic stresses-including fungal pathogens [2, 3], insect herbivory [4], drought [5], salinity [6], and heavy metal toxicity [7], without compromising host growth. Despite this agricultural potential, the association is typically host-specific [8, 9], limiting its broader application in crop improvement. Expanding the utility of *Epichloë* symbionts will require a deeper mechanistic understanding of how these fungi establish symbiosis and confer stress tolerance-aspects that remain largely unresolved.

Fungal colonization of plants, whether by pathogens or mutualists, is critically dependent on secreted proteins (SPs) that modulate host physiology to facilitate persistence within plant tissues [10]. In pathogenic interactions, SPs suppress host immunity and reprogram metabolism to promote infection. Similarly, mutualistic fungi must finely tune host defense responses to achieve stable colonization, a process in which secreted effectors play key roles. For instance, *Efe-afpA*, a secreted protein identified in the apoplast of *E. festucae*-colonized red fescue, exhibits antifungal activity against plant pathogens and may be required for symbiosis [2, 11]. Although hundreds of putative SPs have been predicted in *Epichloë* genomes, functional characterization has been reported for only a single protein to date, representing a critical gap in our understanding of the molecular basis of *Epichloë*-grass symbioses [12].

Progress in functional genetic studies of *Epichloë* has been further constrained by the lack of efficient transformation systems. As slow-growing filamentous fungi, *Epichloë* species are recalcitrant to conventional protoplast-based methods developed for other fungi [13, 14]. Establishing a high-efficiency genetic transformation system is therefore a prerequisite for accelerating functional genomics and dissecting the molecular mechanisms underlying *Epichloë*-grass interactions.

In this study, we investigated the symbiosis between *Epichloë gansuensis* and drunken horse grass (*Achnatherum inebrians*), a well-characterized system in which endophyte colonization markedly enhances host drought adaptation [15, 16]. Through transcriptomic profiling during host colonization, we identified *EgSPE*, a gene encoding a secreted protein that is strongly induced in planta, suggesting a potential role in symbiotic interaction. To enable functional analysis, we established a substantially improved genetic transformation system for *Epichloë*, incorporating optimized culture conditions and a split-marker deletion strategy. Using this platform, we demonstrate that EgSPE is important for normal fungal growth and efficient host colonization, and that it contributes critically to endophyte-mediated resistance to water deficit. These findings provide new insight into the molecular mechanisms by which *Epichloë* endophytes promote abiotic stress tolerance in grasses.

## Results

### Expression of the secreted protein EgSPE in *E. gansuensis* is highly induced during initial host colonization

**Fig. 1:**
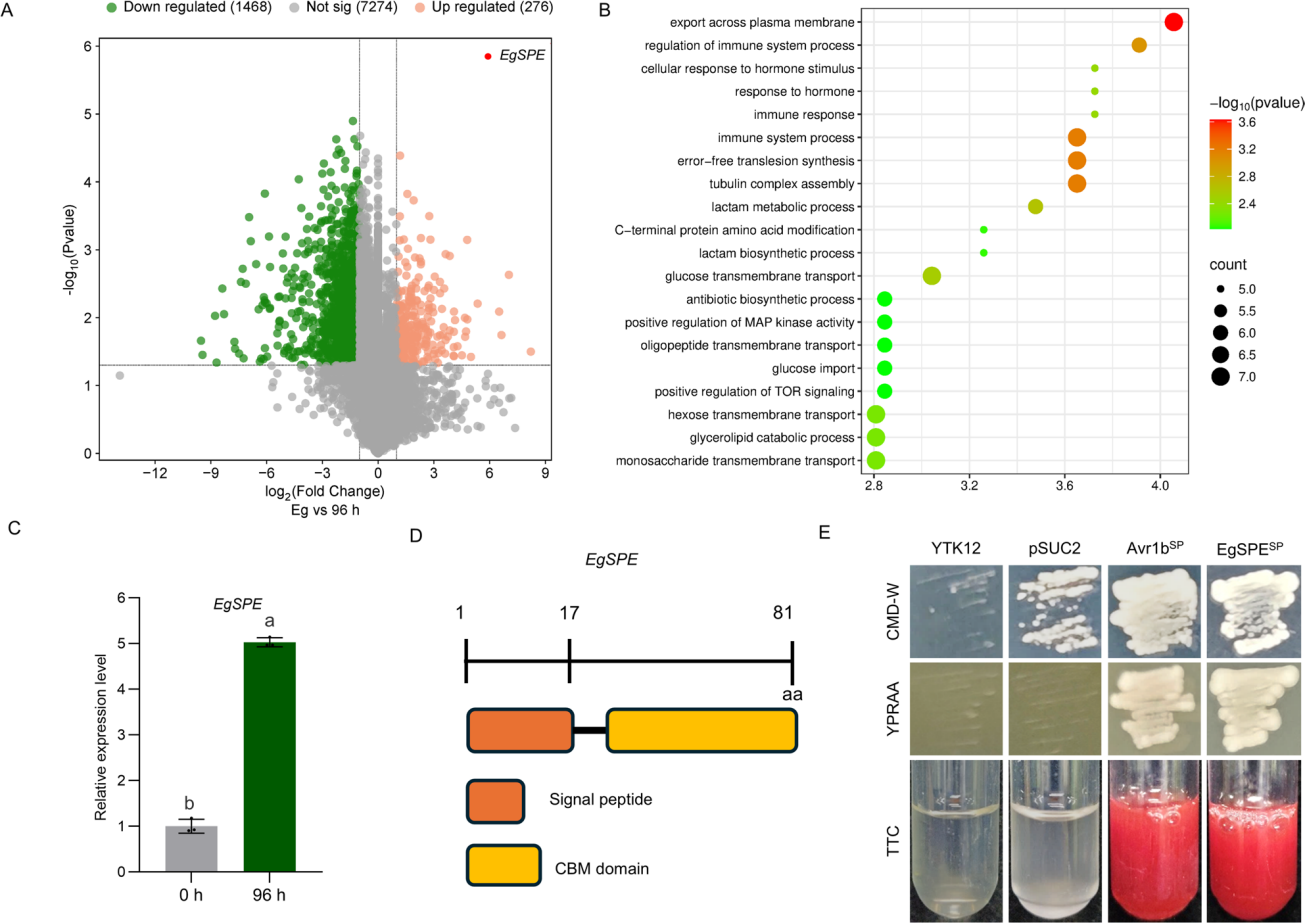
Expression of a secreted protein EgSPE in *E. gansuensis* is highly induced during host colonization. **A** Volcano plot displaying differentially expressed genes between *E. gansuensis* in culture versus inoculated into *A. inebrians* at 96 hpi. Genes with a |log₂FC| > 1 are highlighted: up-regulated in orange, down-regulated in green. **B** Gene ontology (GO) analysis of differential proteins in *E. gansuensis* biological process. Number of proteins in each GO terms indicated. The top 20 significantly enriched pathways for each strain are displayed, with circle size representing enrichment ratio and color indicating −log10 (*P*-value < 0.05). **C** Relative expression levels of EgSPE were analyzed in inoculated with *E. gansuensis* 0 and 96 hpi. The values represent the average of three biological replicates. Different letters indicate significant differences at P≤0.05 (one-way ANOVA, Tukey post-test, n=3, three independent experiments). **D** Schematic representation of EgSPE and the position of the signal peptide. **E** The secretion function of the predicted signal peptides was proved by employing a yeast secretion system with *Saccharomyces cerevisiae* strain YTK12. pSUC2 carrying signal peptides from EgSPE and the positive control Avr1b could all make YTK12 survive on CMD-W and YPRAA medium, while YTK12 containing empty pSUC2 as a negative control failed to grow on YPRAA

In order to explore the molecular mechanism of *E. gansuensis* colonization in *A. inebrians*, we performed transcriptomic analysis of the *A. inebrians*-*E. gansuensis* interaction at 0 and 96 h post-inoculation (hpi). Differential expression analysis revealed 276 significantly upregulated and 1,468 downregulated fungal genes during host infection (Fig. 1A). Functional enrichment analysis showed that upregulated genes were primarily associated with transmembrane export, immune regulation, DNA repair, and tubulin assembly (Fig. 1B). Among these, a gene encoding a secreted protein, designated *EgSPE* (*E. gansuensis* effective secreted protein), exhibited the most significant induction following host inoculation (Fig. 1A).

We further validated EgSPE expression under colonization. *EgSPE* transcript levels were markedly elevated during fungal inoculation (Fig. 1C). Structural analysis predicted that EgSPE contains an N-terminal signal peptide and a carbohydrate-binding module (CBM) family 19 domain (Fig. 1D). We then validated the functional secretion of the predicted signal peptide via a yeast signal sequence trap assay (Fig. 1E). These results demonstrate that the N-terminal signal peptide effectively directs protein secretion in vivo, supporting the classification of EgSPE as a functional protein during the initial infection phase.

### EgSPE is required for mycelial growth of *E. gansuensis*

To functionally characterize EgSPE, an efficient genetic manipulation system for *Epichloë gansuensis* was required. Conventional methods are largely ineffective in *Epichloë* species due to slow growth and poor protoplast yield, as 15-day-old mycelia yielded only ~ 1.8 × 10⁶ protoplasts g⁻¹ with incomplete cell wall digestion (Fig. S1A, B;Fig. S2A). To overcome this, we redesigned the culture strategy to obtain younger mycelia (4 d on cellophane-overlaid PDA followed by 4 d in YPD liquid medium; Fig. S2B), which improved protoplast yield to 6.9 × 10⁸ g⁻¹ with complete digestion and increased transformation efficiency nine-fold, yielding > 175 transformants µg⁻¹ DNA (Fig. S2A-E). Using this system, we generated both EgSPE overexpression and knockout mutants, achieving a ~ 70-fold elevated transcript level in an overexpression line and a 12% knockout frequency for Δ*egspe* mutants (Fig. S2F, G; Fig. S3). Our mutant identification failed to confirm single-locus disruption, which is a limitation. In future experiments, whole-genome sequencing should be employed to further verify single-locus integration. This optimized platform thus enables efficient genetic manipulation of *E. gansuensis* and provides a foundation for dissecting the molecular mechanisms of *Epichloë*-grass symbiosis.

**Fig. 2:**
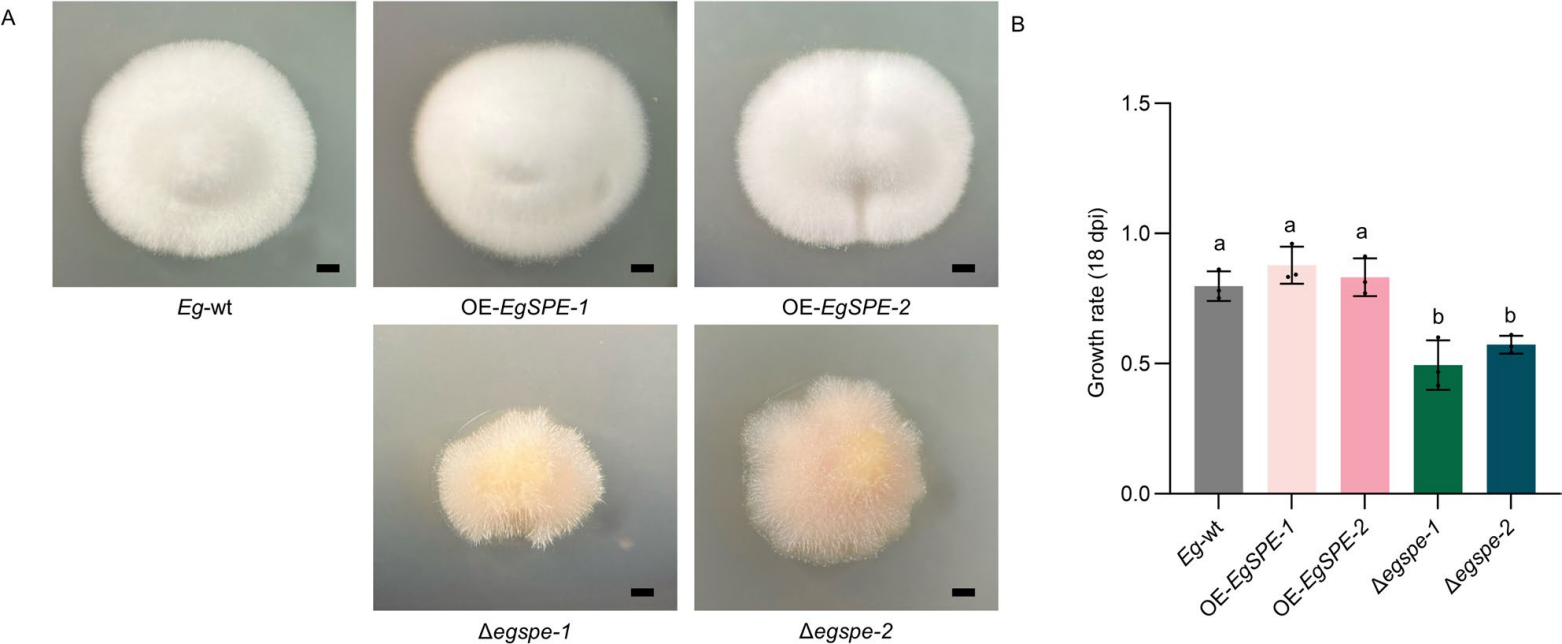
EgSPE is required for mycelial growth of *E. gansuensis*. **A** Colony morphology of wild-type, overexpressed and knockout strains on PDA medium, 22°C, dark culture for 18 d, Scale bars = 1 mm. **B** Colony growth rate on day-18 of growth on PDA. Data shown are means ± SD (n=3, three biological replicates per treatment), Different letters indicate significant differences at *P*≤0.05 (one-way ANOVA, Tukey post-test)

To determine whether EgSPE contributes to fungal development, we examined colony morphology and radial growth of overexpression and knockout strains in comparison with the wild type (*Eg*-wt) on PDA medium. While overexpression lines (OE-*EgSPE*) displayed growth characteristics indistinguishable from the *Eg*-wt, knockout mutants (Δ*egspe*) exhibited reduced colony size, altered pigmentation and significantly slower radial expansion on both hygromycin-containing and antibiotic-free media (Fig. 2A, B; Fig. S4). These results indicate that EgSPE is critical for maintaining normal vegetative growth and mycelial morphogenesis in *E. gansuensis*.

### EgSPE is critical for early fungal proliferation and systemic host colonization

**Fig. 3:**
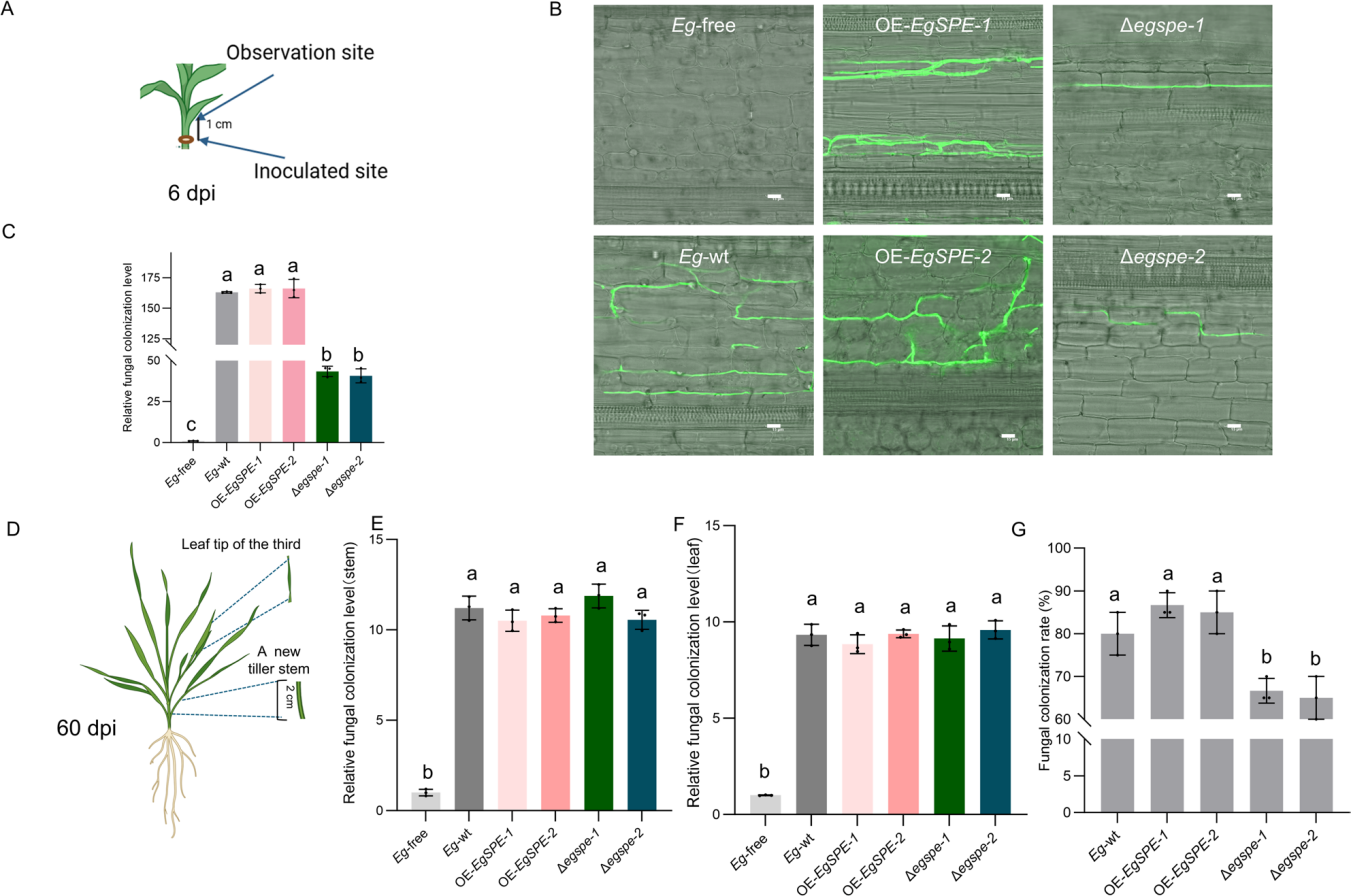
Early fungal proliferation and systemic colonization require EgSPE. **A** Diagram of endophytic fungal inoculation and sample collection (6 dpi). **B** Micrographs of *A. inebrians* stems inoculated with wild-type, OE-*EgSPE*, and Δ*egspe* strains of *E. gansuensis* to show the progression of tissue invasion. Samples were collected at 6 dpi and stained with WGA-AF488. Scale bars = 15 µm. **C** Fungal biomass was quantified in symbiotic samples collected 1 cm above the original inoculation site at 6 dpi. The elongation factor gene was used as the reference gene (*qFactor*-F/R). The values represent the mean of three biological replicates. The *idtG* gene was used as the target gene to quantify *E. gansuensis* colonization. **D** Diagram of endophytic fungal inoculation and sample collection after 60 dpi. **E** Fungal biomass in newly emerged tiller stems at 60 dpi. **F** Fungal biomass in the tips of the third leaves at 60 dpi. Data are means ± SD (n=3, three biological replicates per treatment). **G** Endophytic fungal hyphal colonization rate. Inoculation of Eg-wt, OE-*EgSPE* and Δ*egspe* into *A. inebrians* stems, and collect samples for PCR identification 60 d later. Different letters indicate significant differences at *P*≤0.05 (one-way ANOVA, Tukey post-test)

To determine the role of EgSPE in host-fungus interactions, we monitored fungal invasion in *A. inebrians*. At 6 days post-inoculation (dpi), early hyphal expansion was assessed in tissues approximately 1 cm above the inoculation site (Fig. 3A). Quantitative biomass analysis and WGA-AF488 fluorescence staining revealed that, while OE-*EgSPE* strains exhibited proliferation levels comparable to those of the wild-type (*Eg*-wt), the Δ*egspe* mutants were significantly impaired in their ability to invade host tissues, showing a marked reduction in fungal biomass (Fig. 3B, C). To determine whether EgSPE affects further endophyte extension, we measured fungal biomass in the tip of the third leaf and in newly emerged, non-inoculated tillers of 60-dpi plants (Fig. 3D). Hyphae in these tissues were less abundant, but no significant difference was observed between the mutants and the *Eg*-wt (Fig. 3E, F), indicating that EgSPE attenuates, but does not abolish, hyphal expansion capacity in the host plant.

However, the successful inoculation rate was affected by the presence of EgSPE. At 60 dpi, the colonization frequency of inoculated plants was assessed. OE-*EgSPE* did not significantly increase the colonization rate, whereas the Δ*egspe* mutants caused a pronounced reduction compared with *Eg*-wt (Fig. 3G). Collectively, these results demonstrate that EgSPE promotes both early fungal proliferation in host tissues and subsequent systemic colonization in the *E. gansuensis*-*A. inebrians* symbiosis.

### EgSPE activates host drought responses and enhances drought tolerance in *A. inebrians*

The *E. gansuensis-A. inebrians* symbiosis is known to enhance host tolerance to multiple environmental stresses, particularly drought [16]. Consistent with this role, *EgSPE* expression was not only induced during endophyte-host interaction but further upregulated PEG‑induced osmotic stress conditions (Fig. 4A). Heterologous expression of EgSPE in *N. benthamiana* significantly enhanced the expression of the drought-responsive marker gene *RD29A* under PEG treatment, suggesting a potential role of EgSPE in endophyte-mediated drought tolerance (Fig. 4B, C).

**Figure:**
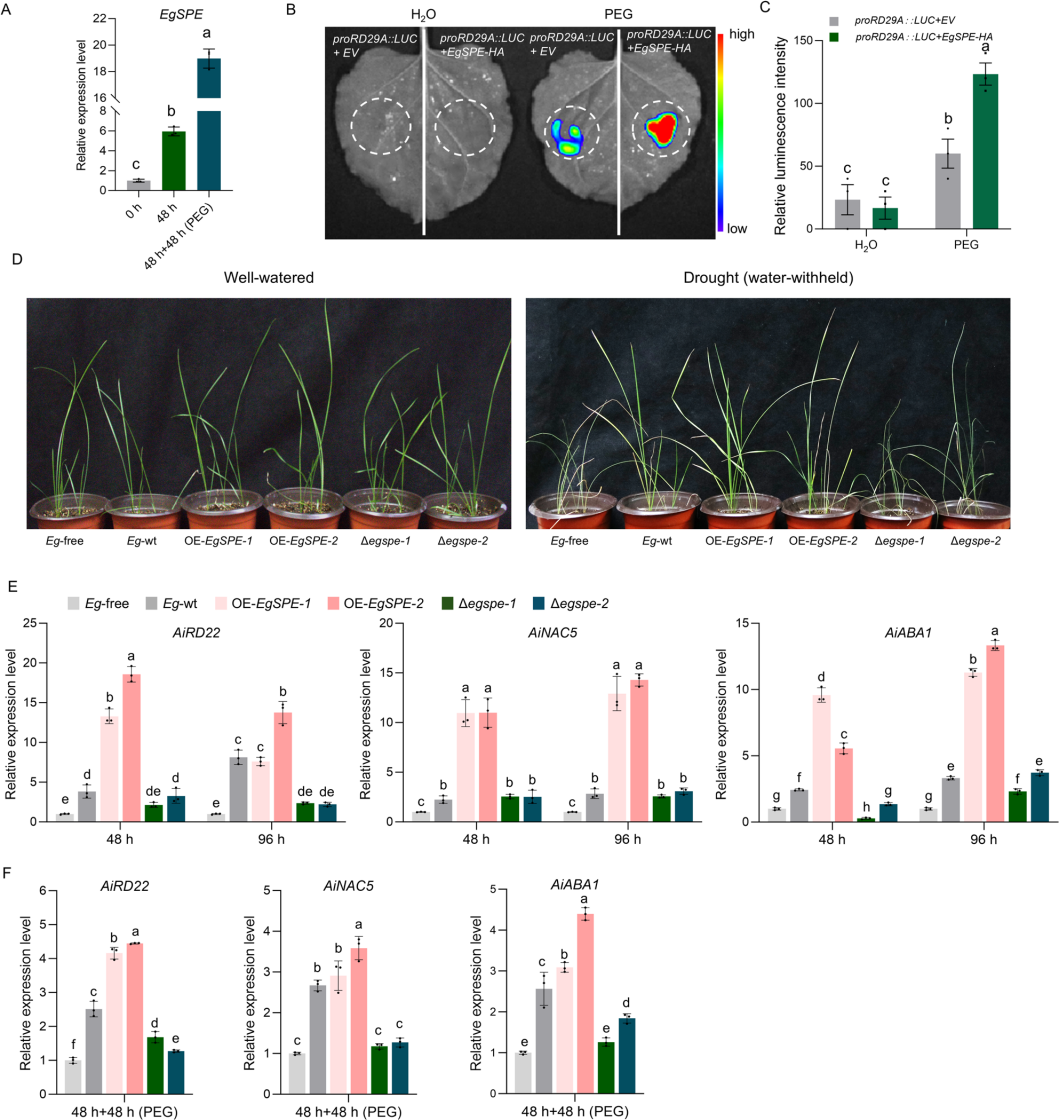
EgSPE activates host drought responses and enhances drought tolerance in *A. inebrians*. **A** Expression levels of *EgSPE* were analyzed in 20% PEG6,000 treatment for 48 h after 48 h of inoculation). **B** Luminescence after treatment with water (left) and 20% PEG6,000 (right) for 8 h. The color scale at right shows the luminescence intensity from dark blue (lowest) to red (highest). **C** Quantitation of the luminescence intensities shown in (B) was measured using the Image J software. **D** *A. inebrians* drought phenotype. *Eg*-free, inoculated *Eg*-wt, OE-*EgSPE* and Δ*egspe* after 60 d before treatment (left) and after drought (water-withheld) (right). **E** RT-qPCR was used to verify the expression of downstream genes responsed to drought response. *Eg*-free, inoculated *Eg*-wt, OE-*EgSPE* and Δ*egspe*, collected separately at 48 h after inoculation, inoculated for 96 h (water treatment for 48 h after 48 h of inoculation). **F** 20% PEG6,000 treatment for 48 h after 48 h of inoculation. Data are means ± SD (n=3, three biological replicates per treatment), Different letters indicate significant differences at P≤0.05 (one-way ANOVA, Tukey post-test).

To further confirm the functional role of EgSPE in the native host, we assessed drought responses of *A. inebrians* colonized by different *E. gansuensis* strains after verifying successful colonization via fungal marker detection (Fig. S5A, B). Under 15 d water-withheld treatment, plants inoculated with the wild-type and OE-*EgSPE* strains exhibited significantly enhanced drought tolerance compared with *Eg*-free plants. In contrast, plants colonized by Δ*egspe* mutants exhibited a similar phenotype to *Eg*-free plants, conferring no significant drought resistance. (Fig. 4D).

We further analyzed the expression of downstream drought-responsive genes including *AiRD22*, *AiNAC5*, and *AiABA1*. At 48 and 96 h post-inoculation (hpi) without PEG treatment, all tested *E. gansuensis* strains (*Eg*-wt, OE-*EgSPE*, and Δ*egspe*) induced higher expression levels of these genes in *A. inebrians* compared with *Eg*-free plants. Notably, OE-*EgSPE* colonization resulted in significantly stronger upregulation than both the *Eg*-wt and Δ*egspe* (Fig. 4E). Crucially, under PEG treatment (48 hpi followed by 48 h PEG exposure), marked differences were observed among the colonized plants. *Eg*-wt and OE-*EgSPE* colonized plants both exhibited significantly enhanced expression of *AiRD22*, *AiNAC5*, and *AiABA1* compared with *Eg*-free plants, with OE-*EgSPE* again showing the strongest induction. In sharp contrast, plants colonized by the Δ*egspe* mutants showed only marginal or no detectable induction of these genes under the same PEG treatment (Fig. 4E, F). These results demonstrate that EgSPE is important for the activation of host drought-responsive pathways under PEG‑induced osmotic stress conditions. Together, these findings indicate that EgSPE plays a critical role in *E. gansuensis*-mediated enhancement of drought tolerance in *A. inebrians*, a process associate with the upregulation of key drought-responsive genes under water-limited conditions.

## Discussion

*Epichloë* endophytic fungal symbionts are widely recognized for conferring enhanced stress tolerance to their host plants [17–19]. Notably, these endophytes improve drought resistance in grasses without compromising host growth, a trait of considerable value for developing more resilient crops and forages under the pressures of climate change. Understanding the molecular mechanisms underlying *Epichloë*–grass interactions is therefore critical for the efficient and widespread application of this endophyte genus. In this study, we identified a secreted protein (SP) from *E. gansuensis*, EgSPE, which plays multiple critical roles in *E. gansuensis* and its interaction with host grass, including the regulation of fungal morphology and growth rate, the establishment of systemic symbiosis, and the mediation of host drought tolerance. This SP serve as a useful entry point for elucidating the molecular crosstalk mechanisms in *Epichloë*-grass interactions.

*EgSPE* encodes a small protein of 81 amino acids, contains a single carbohydrate-binding module (CBM) domain (Fig. 1D). CBMs are non-catalytic domains that typically enhance polysaccharide-degrading efficiency by targeting enzymes to their substrates and disrupting substrate structure; they also participate in diverse biological processes-including pathogen defense and biosynthesis-through specific carbohydrate recognition [20]. For instance, LysM (CBM50) effectors such as Ecp6, Slp1, and RsLysM sequester chitin oligomers in the apoplast, competing with plant chitin receptors like CEBiP to prevent chitin perception and thereby dampening ROS bursts and defense-related gene expression [21]. The CBM domain in EgSPE belongs to the CBM 19 domain family which previously is characterized as a chitin-binding module in fungal chitinases [22]. Chitin is a canonical elicitor of plant immune responses during fungus infection. Suppressing these immune responses are main functions of fungal SPs involved in microbial colonization [23]. The phenotypic alterations observed in the Δ*egspe* mutants suggest that EgSPE contributes to fungal morphogenesis and symbiotic performance (Figs. 2 and 3). Whether these function of EgSPE due to its capability in modulating plant immune responses needs further investigation.

Emerging evidence shows that effectors from beneficial fungi can do more than suppress host immunity: they also promote plant growth and enhance stress tolerance. For instance, effectors from *Serendipita indica* modulate phytohormone signaling to stimulate growth [24], and the effector SIE141 targets CDSP32 to activate NPR1, thereby boosting resistance to both *Phytophthora* and salt stress [25]. EgSPE induces the drought-responsive marker *RD29A* in *N. benthamiana* under PEG treatment and is essential for upregulating host drought-responsive genes (*AiRD22*, *AiNAC5 AiABA1*) in the native grass symbiosis under water deficit. The ability of EgSPE to activate drought responses in both tobacco and its natural host suggests functional conservation across plant species. Certain CBM-containing proteins have been reported to enhance ROS bursts and contribute to drought tolerance [26]. Thus, EgSPE represents a promising candidate for dissecting the molecular mechanisms of endophyte-mediated drought adaptation and for developing new strategies to improve crop drought resistance.

We analyzed the conservation of EgSPE in endophytic fungi. EgSPE homologs were identified in the predicted secretomes of two additional *Epichloë* species, *E. sibirici* and *E. scottii*, with the *E. sibirici* homolog showing 100% identity and 100% query coverage. Public BLASTP searches further detected EgSPE-like proteins in several fungal taxa with reported endophytic or endophyte-capable lifestyles, including the *Akanthomyces/Lecanicillium* complex, *Purpureocillium*, *Trichoderma*, *Metarhizium*, *Beauveria* and *Pochonia* (Table S1). Because most homologs were annotated as hypothetical or uncharacterized proteins, we did not infer that they represent experimentally validated effectors. Nevertheless, the presence of EgSPE homologs across diverse endophytic fungal lineages suggests that this protein may represent a conserved secreted component associated with plant colonization and symbiotic adaptation. Whether these homologs share functional similarity with EgSPE in promoting plant colonization or stress tolerance remains to be investigated.

The identification and functional characterization of EgSPE highlight the importance of secreted protein. These proteins regulate the growth and development of *Epichloë* endophytes and their symbiotic interaction with host grasses. Our findings support the view that fungal secreted proteins can contribute to multiple aspects of the *Epichloë*-grass association, including fungal growth, host colonization, and endophyte-mediated stress tolerance. Future studies should aim to identify additional effector or secreted proteins in *Epichloë* and determine their secretion, host perception and downstream effects in host plants. The optimized fungal transformation system developed in this study will facilitate functional genetic analyzes and accelerate the discovery of symbiosis-associated genes in *Epichloë*.

## Conclusion

In this study, we identify EgSPE as a secreted protein from *E. gansuensis* that contributes to fungal growth, early symbiotic establishment, and endophyte-mediated drought tolerance in *A. inebrians*. *EgSPE* deletion reduced fungal growth and early colonization and was associated with weakened host drought-responsive gene expression and reduced drought tolerance. Together, these findings suggest that EgSPE participates in multiple aspects of the grass-endophyte association and provide a foundation for further mechanistic studies of stress adaptation in symbiotic systems.

## Materials and methods

### Plant material and growth conditions

The seeds of the drunken horse grass (*Achnatherum inebrians*) were used as test material, which is collected from Qinghai-Tibet Plateau, China (97.27°E, 30.24°N). A sufficient amount of the grass seeds was disinfected with 75% ethanol for 1 min, then treated with 2% sodium hypochlorite for 30 min, and finally rinsed with sterile water 5 times. The seeds were cultured in ½MS medium and placed in a 23 ℃ climate chamber for growth, with a photoperiod of 16 h of light/8 h of darkness, and a relative humidity of 60%. The seeds were allowed to grow up to 15–20 d.

### *Epichloë gansuensis* culture assay

The fungal strain was isolated from *A. inebrians* and identified as *E. gansuensis*, exhibiting 99% sequence similarity to strain e7080 (NCBI Taxonomy ID: 447254) from the National Center for Biotechnology Information (NCBI). The endophyte was cultured on potato dextrose agar (PDA) medium (Coolaber, cat. no. PM0520-250 g) in an artificial climate chamber (MGC-450HP-2, Bluepard) at 22 °C under dark conditions. Before optimization, mycelia from the colony edge were transferred to 250 mL Erlenmeyer flasks, each containing 100 mL of yeast peptone dextrose medium (YPD), and incubated at 22 °C with shaking at 200 rpm under dark conditions for 15 d. After optimization, mycelia from the colony edge were transferred to 500 µL of YPD medium and homogenized using a ball mill at 30 m/s for 30 s until fully lysed. A 200 µL aliquot of the homogenate was spread onto PDA medium overlaid with cellophane membranes and incubated at 22 °C in darkness for 4 d. Fresh mycelia were scraped from the cellophane membranes and transferred to 500 µL of YPD medium. The mixture was homogenized using a ball mill at 30 m/s for 30 s until fully lysed. The homogenate was then inoculated into four 250 mL Erlenmeyer flasks, each containing 100 mL of YPD medium, and incubated at 22 °C with shaking at 200 rpm under dark conditions for 4 d.

### Protoplast isolation of *E. gansuensis*

The fungal cultures were divided into batches and transferred to sterilized 50 mL centrifuge tubes. The tubes were then centrifuged at 4 °C and 5,000 × g for 10 min. The pellet was washed three times with 0.7 mol/L NaCl, followed by final centrifugation at 4 °C and 5,000 × g for 5 min, after which the supernatant was discarded. In case of enzymatic digestion, 0.2 g of fungal biomass was suspended in 1 mL of enzyme solution (prepared in 0.7 mol/L NaCl: 2.0% lysing enzymes from *Trichoderma harzianum* (Sigma-Aldrich, cat.no.L1412-5G), 1.5% Driselase basidiomycetes (Sigma-Aldrich, cat.no.D9515-1G), 1.0% snailase (Sangon Biotech, cat.no.A600870-0001), 1.0% cellulase (Sangon Biotech, cat.no.A00602610-0001), and 3 mg/ml Bovine serum albumin (Sigma-Aldrich, cat.no.A1933-5G)). The enzyme solution was filter-sterilized using a 0.45 μm filter column. The mixture was incubated in a water bath at 32 °C for 4–5 h.

The digested suspension was filtered through two layers miracloth (pre-wetted with 0.7 mol/L NaCl) into a 50 mL centrifuge tube. To the filtrate, 1 mL of STC buffer (1.2 M sorbitol, 10 mM Tris-HCl, 50 mM CaCl₂, pH 7.5) was added, and the mixture was centrifuged at 4 °C and 3,000 × g for 20 min. The supernatant was discarded, and the pellet was resuspended in 1 mL of STC buffer, followed by centrifugation at 4 °C and 2,000 ×g for 10 min. This washing step was repeated once. The final protoplasts pellet was gently resuspended in 500 µL of STC buffer. Protoplast concentration was quantified using a hemocytometer under a microscope, and the suspension was diluted with STC buffer to achieve a final density of ≥ 5 × 10⁶ protoplasts per mL.

### EgSPE knockout and overexpression vector construction

The overexpression vector was constructed using the plasmid backbone pPCT74. To facilitate detection, an HA epitope tag was incorporated into the construct. The target gene was placed under the control of the *ToxA* promoter, a strong constitutive promoter in fungi, to ensure high expression levels. The native termination codon of the gene was removed, and an HA tag was fused to the C-terminal end of the protein, enabling efficient detection via PCR methods.

The strategy based on the split-marker approach was used to obtain *EgSPE* gene knockout strains. In the obtained target gene sequence, an upstream primer was designed approximately 1,000 bp before the start codon (excluding the start codon), with the 5’ end of the upstream primer incorporating a hygromycin linker homologous arm. A downstream primer was designed approximately 1,000 bp after the stop codon (excluding the stop codon), with the 3’ end of the downstream primer incorporating a hygromycin linker homologous arm. The fused product was then purified and used as a template for amplification to obtain the knockout fragment. Additionally, we employed a strategy using hygromycin as the homologous arm, amplifying a fragment from approximately 1,000 bp before the start codon to the first two-thirds of the hygromycin sequence, and another fragment from the last two-thirds of the hygromycin sequence to approximately 1,000 bp after the stop codon. The overlap between the two truncated *hph* gene fragments was 500–700 bp [27].

### PEG-mediated protoplast transformation of *E. gansuensis*

The *E. gansuensis* PEG-mediated protoplast transformation was performed based on the method reported by [28] with some modifications. For optimization of the *E. gansuensis* protoplast PEG transformation method, using 80 µL of protoplasts was mixed with 1 µg (5 µL) of linearized plasmid (overexpression and knockout fragments), then added 5 µL of 50 mM spermidine. Subsequently, added 90 µL of 40% PEG4,000 and stirred thoroughly, incubated at room temperature for 10 min. Then added 2 volumes of STC (360 µL), gently stirred to mix, centrifuged at 4 °C and 2,000×g for 5 min and repeated the centrifugation step three times. Finally, retained 100 µL of STC and gently resuspended the pellet by flicking.

### Protoplast regeneration and screening for stable transformants of *E. gansuensis*

A 100 µL of the transformation product was spread onto regeneration medium (PDB, 0.6 M sucrose, and 1.0% agar). Hygromycin (100 µg/mL) (Solarbio: H8080) was used to screen the overexpression transformants and knockout transformants. Untransformed protoplasts were spread onto regeneration plates with and without antibiotics, followed by incubation at 22 °C in the dark for 7–10 d. Then, the grown transformants were transferred to fresh antibiotic-resistant medium and DNA was extracted for identification (Fig. S3A). The *Hyg*-F/R primer pairs were employed to detect the introduction of the hygromycin gene, *EgSPE*-F/R to verify gene knockout, and *EgSPE*-outF/R to confirm whether the target gene was replaced by hygromycin (Fig. 3SB). The PCR band sizes were used to determine the results (Fig. S3C-E).

### *E. gansuensis* growth rate measurement assay

An inoculation pipette of 0.5 mm was used to inoculate the wild-type, overexpressed and knockout strains onto PDA plates and cultured in darkness at 22 ℃. The wild-type *E. gansuensis* was inoculated onto an antibiotic-free medium, and the overexpressed and knockout strains were inoculated onto a medium containing hygromycin (100 µg/mL) (Solarbio: H8080). Growth rate was assessed on both hygromycin‑containing and antibiotic‑free PDA plates. The colony diameter was recorded every three days using a vernier caliper, and measured continuously for 30-d period using the cross-crossing method. Each strain had three biological replicates, and the experiment was repeated three times. The growth rate was fastest on the 18th day.

### Fungal colonization rate and relative expression measurement assay

The *E. gansuensis* detection of endophytic fungal colonization rate was performed based on the method reported by [29] with some modifications. For sterile-cultured young seedlings of drunken horse grass for 15–20 d, the seedlings were inoculated by making an incision about 1 cm above the root and then inserting mycelia into the area of incision. Control seedlings were inoculated with agar. The inoculated seedlings were maintained in a growth chamber at 24℃ in the dark for 6 d, followed by a 12/12 h light/dark regimen for 6 d, following the procedures reported in [30]. Six days after inoculation, we collected samples 1 cm above the inoculation site, extracted RNA, and performed relative quantification of endogenous fungi. Further, the seedlings were transplanted into plastic pots containing heat-sterilized vermiculite (150℃ for 30 min) and maintained in a growth chamber (16/8 h light/dark regime, 24℃). The colonization levels of endophytic fungi 60 dpi, collect samples of a new tiller stem and the third leaf tip, extracted RNA from plant tissues at the same location for RT-qPCR.

After 60 dpi, the colonization rate of endophytic fungi in drunken horse grass was assessed for each transgenic strain treatment. cut off a tiller and trim about 2 cm of the stem from the root upwards, and extract DNA for subsequent experiments. Fungal colonization was detected by PCR amplification of the indole alkaloid biosynthesis gene *idtG*. A total of 60 samples were analyzed per treatment, every 20 samples constitute one replicate, and each sample has 3 replicates.

### RT-qPCR determination of gene expression

Reverse Transcription Quantitative Polymerase Chain Reaction (RT-qPCR) was used to determine the expression level of the target gene of the overexpressed strain. RNA was extracted from a single (transformed) mycelium colony, reverse transcribed into cDNA, diluted to an appropriate concentration, and designed specific primers for the target gene with extension factor as the internal reference gene. ABclonal SYBR Green (RT-qPCR reagent No. RK21203) was used to perform RT-qPCR for the overexpressed transformed strain *E. gansuensis*, all samples were analyzed with three technical replicates. The RT-qPCR instrument automatically generated amplification and melting curve, through which the Ct value of target gene and internal reference gene in each sample was determined (Ct, cycle threshold, the number of cycles when the fluorescence signal reached the set threshold). Relative gene expression was calculated using the 2^−△△^Ct method [31].

### Transcriptome analysis

The total RNA from inoculated stems with different time points inoculated and control was individually extracted to construct the RNA-seq library. Three independent biological replicates were performed for each condition. Each replicate originated from separate plant stems, with RNA extraction, library preparation, and sequencing carried out independently. These clean reads were then aligned to the reference genome of *Epichloë gansuensis* (e7080) using HISAT2 software, and the alignment files were sorted using Samtools [32, 33]. Sequencing was performed using Illumina NovaSeq6,000 platform. DESeq2 (V1.22.2) /edgeR (V3.6.8) was used for differential expression significance analysis, and the screening threshold was FDR (false discovery rate) < 0.05, log_2_FC (fold change) > 1 or < -1. Gene Ontology (GO) annotation was performed using the eggNOG-mapper web server (http://eggnog-mapper.embl.de/). Significantly enriched GO terms were identified with a Bonferroni-corrected *P*-value threshold of ≤ 0.05, using the whole transcriptome as the background. Final gene function annotations were obtained by aligning the genes to the UniProt and non‑redundant (NR) protein sequence public databases.

### *N. benthamiana* transient expression assay

The genetic screen used the firefly luciferase reporter gene driven by the drought responsive *RD29A* promoter (*RD29A::LUC*) [34]. In *N. benthamiana*, each leaf was divided into two halves, with *proRD29A::LUC* co-expressed alongside EV on one side and EgSPE on the other. After 24 h of expression, the experimental group was treated with 20% PEG6,000 for 8 h, water treatment as a control. Leaves were cut and sprayed with D-Luciferin (GlpBio: GC11860) on the underside of the leaves, then observe and photographed using the Living Imaging system (PerkinElmer IVIS Lumina III).

### WGA-AF488 staining to observe fungal colonization assay

To observe endophytic fungal colonization, sterilely cultured *A. inebrians* seedlings were inoculated. Under a dissecting microscope, a 2–3 mm longitudinal slit was cut at the developing apical meristem between the mesocotyl and coleoptile using a sterile scalpel. A small piece (approximately 2 mm × 2 mm) of freshly grown mycelium taken from the colony edge was inserted into the slit. The seedlings were then incubated on ½ MS plates at 22 °C in the dark for an additional 6 d [35]. At 6 dpi, tissue segments (extending 1 cm above the inoculation site) were collected for assessment of fungal colonization. The samples were processed for fluorescence imaging according to the staining protocol described by [36], Wheat Germ Agglutinin-Alexa Fluor 488 (WGA-AF488) was used to stain fungal hyphae. Observation was carried out on a Leica SP8 laser scanning confocal microscope, with excitation/emission settings of 488/500–530 nm for eGFP (WGA-AF488).

### Validation of the secretion function of the predicted signal peptide of EgSPE

The yeast signal sequence trap assay used the pSUC2T7M13Ori (pSUC2) vector, which carries a truncated SUC2 gene lacking the initiating methionine and the native signal peptide [37]. The secretion activity of the EgSPE signal peptide was verified. DNA fragments of the signal peptide were synthesized by SynbioB and inserted into pSUC2 through EcoRI and XhoI restriction sites, fused in-frame with the invertase gene [38]. Using the lithium acetate method, 20 ng of the plasmid was transformed into the enzyme-deficient yeast strain YTK12.

After transformation, yeast cells were plated on CMD-W (minus-tryptophan) plates and transferred to fresh CMD-W plates, then stored at 30 °C. PCR with vector-specific primers was used to confirm the transformation. To assess the secretion of the transformant enzyme, colonies were replicated onto YPRAA plates containing raffinose and lacking glucose (1% yeast extract, 2% peptone, 2% raffinose, and 2 µg/mL antibiotic A). The activity of the transformant enzyme was measured by TTC reduction to insoluble red triphenylformazan. Yeast cultures were inoculated into sucrose medium and incubated at 30 °C for 24 h. The pellets were collected, washed, and resuspended in distilled water. The cultures were incubated with 0.1% TTC at 35 °C for 35 min, followed by an additional 10 min at room temperature, and color change was monitored.

### Statistical analysis

The Statistical Product and Service Solutions (SPSS) software was used to conduct the statistical analyzes. All experimental data were tested by Student’s t-test and Tukey post-test. Prism 10.1.2 software (GraphPad, San Diego, CA, USA) was used to generate figures.

## Supplementary Information

Supplementary Material 1: Table S1. Conservation analysis of EgSPE in endophytic fungi. Table S2. Primer used in this study. Figure S1. Prolonged cultivation results in poor protoplast condition. (A) *E. gansuensis* was cultured in YPD liquid medium at 22°C with shaking at 200 rpm under dark conditions for 15 d. (B) *E. gansuensis* in S1A produces protoplasts with incompletely digested cell walls after 4-5 h of enzymatic hydrolysis. Figure S2. Optimized transformation system enables functional analysis of EgSPE. (A) Grow for18 d on PDA medium, 22°C, dark *E. gansuensis*, Scale bars = 1 mm. (B) Grow for 4 d on PDA medium covered with cellophane, 22°C, dark *E. gansuensis*, Scale bars = 10 mm. (C) *E. gansuensis* protoplasts, Scale bars = 10 μm. (D) Protoplasts yield under different treatment methods. YPD (15 d) refers to the cultivation of the YPD liquid medium for 15 d. PDA (4 d) + YPD (4 d) is culturing on a covered cellophane for 4 d, and then crushing the fungi and inoculating them onto YPD liquid medium for 4 d of cultivation. (E) Direct culture for 15 d and PDA (4 d) + YPD (4 d) transformation efficiency. (F) Expression level of different overexpressed strains. (G) Knockout efficiency of split-marker. Data in the figures are means ± SD (n=3, three biological replicates per treatment). **indicates significant differences at *P*≤0.05 (one-way ANOVA, Tukey post-test). Figure S3. Identification of genetically modified strains. (A) The identification of overexpression strains, using wild-type *E. gansuensis* as a negative control, and positive clones were identified using primers *ToxA-F/pCT74jianding-HAR*. (B) Schematic diagram of knockout mutant construction and the primers involved. (C), (D), (E) PCR identification of the knockout mutant. Using wild-type *E. gansuensis *as a negative control, three pairs of primers-*Hyg-F/R, EgSPEout-F/R, and EgSPE-F/R*-were used to ensure the accuracy of the identification results. (F) RT-qPCR determined the expression level of knockout mutants. The elongation factor was used as the reference primer (*qFactor*-F/R). Figure S4. Growth rate of mutants in antibiotic-free medium. (A) Colony morphology of wild-type, overexpressed and knockout strains on antibiotic free PDA medium, 22 °C, dark culture for 10 d, Scale bars = 1 mm. (B) Colony growth rate on day-10 of growth on PDA. Data shown are means ± SD (n=3), Different letters indicate significant differences at P≤0.05 (one-way ANOVA, Tukey post-test). (C) PCR identification of knockout mutants. Figure S5. Identification of successfully inoculated plants before drought treatment. (A). Diagram of endophytic fungal inoculation and sample collection after 60 dpi. (B). PCR identification of positive inoculated fungal. Using the alkali-producing gene unique to *E. gansuensis* as a primer (*idtG-F/R*).

## Data Availability

https://www.ncbi.nlm.nih.gov/bioproject/PRJNA1460266

## Abbreviations

EgSPE: *Epichloë gansuensis* effective secreted protein
OE-EgSPE: EgSPE-overexpressed *Epichloë gansuensis* strain
*Δegspe*: EgSPE knockout *Epichloë gansuensis* strain
*RD29A*: *Responsive to Desiccation 29A*
SPs: Secreted proteins
hpi: hours post-inoculation
dpi: days post-inoculation
CBM: carbohydrate-binding module
GO: Gene Ontology
ROS: reactive oxygen species
MAMP: microbe-associated molecular pattern
PRRs: plant pattern recognition receptors
ABA: abscisic acid
NCBI: National Center for Biotechnology Information
PDA: potato dextrose agar
YPD: yeast peptone dextrose medium
WGA-AF488: Wheat Germ Agglutinin-Alexa Fluor 488
