# Supplementary material for: EgSPE, a secreted protein from Epichloë gansuensis, modulates symbiotic establishment and host drought tolerance
Source: BMC Plant Biol. 2026 May 30;26:1294. doi: 10.1186/s12870-026-09133-1 (PMC13430883; doi:10.1186/s12870-026-09133-1)

**Table S1. Conservation analysis of EgSPE in endophytic fungi**

| **Genus** | **Species** | **ID** | **Name** | **Identity** | **Query coverage** | **Score** |
| --- | --- | --- | --- | --- | --- | --- |
| *Epichloe* | *Epichloe sibirici* | JALAAE010000021.1.112 | NA | 100% | 100% | 167 |
| *Epichloe* | *Epichloe scottii* | CP083248.1.930 | NA | 69% | 75% | 81.6 |
| *Akanthomyces* | *Akanthomyces lecanii* | OAQ96058.1 | hypothetical protein LLEC1_04621 | 56.96% | 94% | 87.8 |
| *Purpureocillium* | *Purpureocillium lilacinum* | XP_018173544.1 | uncharacterized protein PLICBS_009040 | 54.88% | 95% | 85.9 |
| *Trichoderma* | *Trichoderma virens* | XP_013955670.1 | uncharacterized protein TRIVIDRAFT_111979 | 54.43% | 95% | 82.4 |
| *Metarhizium* | *Metarhizium rileyi* | OAA34623.1 | hypothetical protein NOR_08381 | 53.33% | 91% | 81.3 |
| *Pochonia* | *Pochonia chlamydosporia* | XP_018139181.1 | hypothetical protein VFPPC_14578 | 59.49% | 95% | 80.1 |
| *Beauveria* | *Beauveria bassiana* | KGQ02911.1 | hypothetical protein BBAD15_g11861 | 47.37% | 91% | 73.9 |
| *Lecanicillium* | *Lecanicillium* sp. MT-2017a | KAK3190635.1 | hypothetical protein K4F52_003326 | 52.38% | 94% | 68.2 |

| **Name** | **Sequences (5’-3’)** |
| --- | --- |
| *idtG-*F | ATGGCGGCAGATAACTTTCCA |
| *idtG-*R | CCGTGGTTTTATTGCTAAATTGTT |
| *EgSPE-*F | CACCATGAAGTTCGTTGCAGTCACCG |
| *EgSPE-*R | AGCTCTCAAGGGAGGGAACTG |
| *pCT74-EgSPE*F | TGAATGAATATAGGCCAAGCTTATGAAGTTCGTTGCAGTCACCG |
| *pCT74-EgSPE*R | GGTACGTCGTATGGGTAAAGCTTAGCTCTCAAGGGAGGGAACTG |
| *pCT74-HA*F | GACGTACCAGATTACGCTGGGTAAAGCGGCCGCCCGGC |
| *pCT74-HA*R | AAGCTTGGCCTATATTCATTCATTGTCAGC |
| *ToxA-*F | TGGAATGCATGGAGGAGTTCTG |
| *pCT74jianding-HA*R | TTACCCAGCGTAATCTGGTACG |
| *EgSPE-up*F | GACAACGCCAGACGGGAGTCT |
| *EgSPE-up*R | CTTGGCTCATGGAGCCCTTTAG |
| *EgSPE-tef*F | TAAAGGGCTCCATGAGCCAAGTGTGTCTGCCTCTAGAGTGATG |
| *EgSPE-tef*R | GGTGAGTTCAGGCTTTTTCATGACCCTTTGGCTCGCTTAGTCAG |
| *EgSPE-Hyg*F | ATGAAAAAGCCTGAACTCACCGC |
| *EgSPE-Hyg*R | AGTTCTACCATAACTTATCAGCTATTCCTTTGCCCTCGGACGAG |
| *EgSPE-down*F | CTGATAAGTTATGGTAGAACTAGT |
| *EgSPE-down*R | GGTGACTGCAACGAACTTCATG |
| *EgSPE-out*F | CTCACTCCGAGGCAACCGGC |
| *EgSPE-out*R | GCCAGTAGGGTCGTAGTCTCAC |
| *TYB-Hyg*F | TATTGCATCTCCCGCCGTGC |
| *TYB-Hyg*R | TGCAAGCTCCGGATGCCTC |
| *EgSPE-q*F | ACGTACAGGTGCAATAAGGC |
| *EgSPE-q*R | AACTTGCACACAGTCTGACG |
| *qFactor-*F | AAAAAGCAACCGAATGCAAG |
| *qFactor-*R | CGAGACGACATAACTACATGTATCAAA |

**Table S2. Primer used in this study**

**Figure S1. Prolonged cultivation results in poor protoplast condition.**


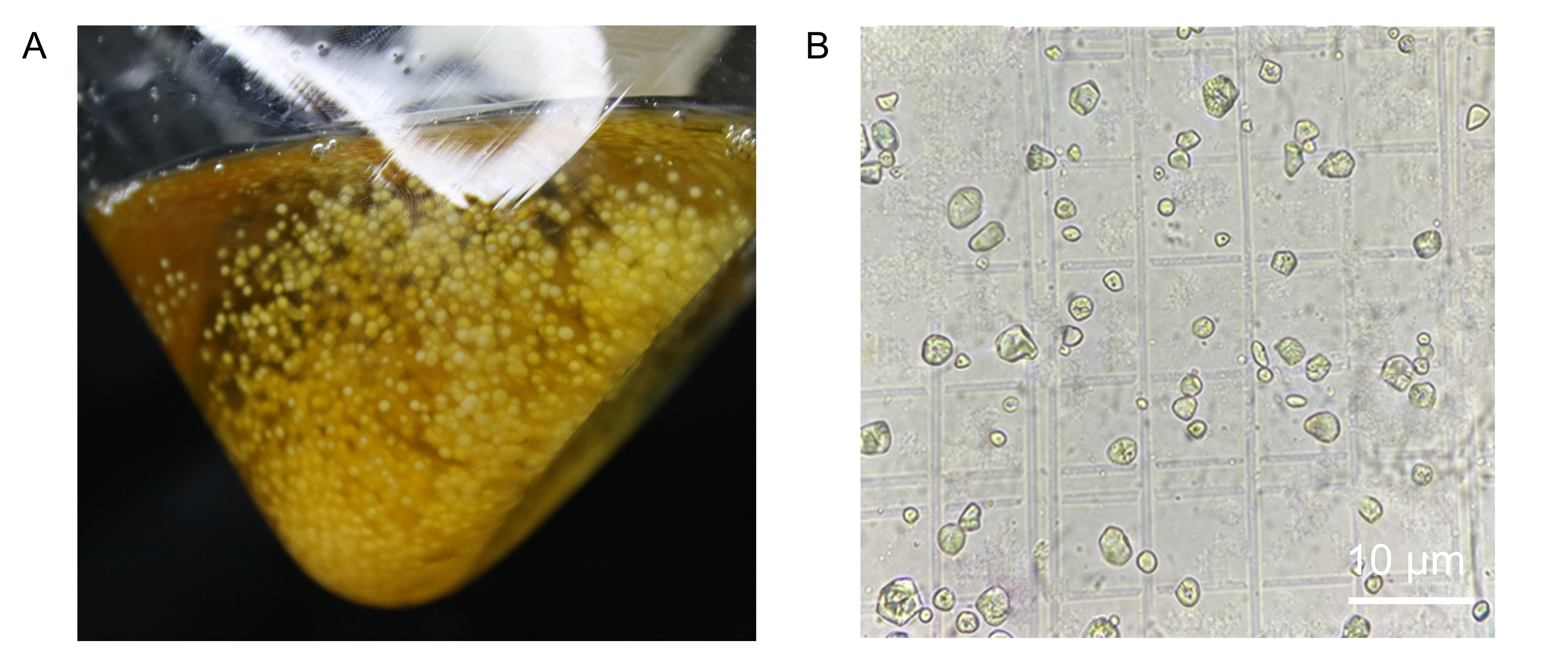


**Figure S2. Optimized transformation system enables functional analysis of EgSPE**


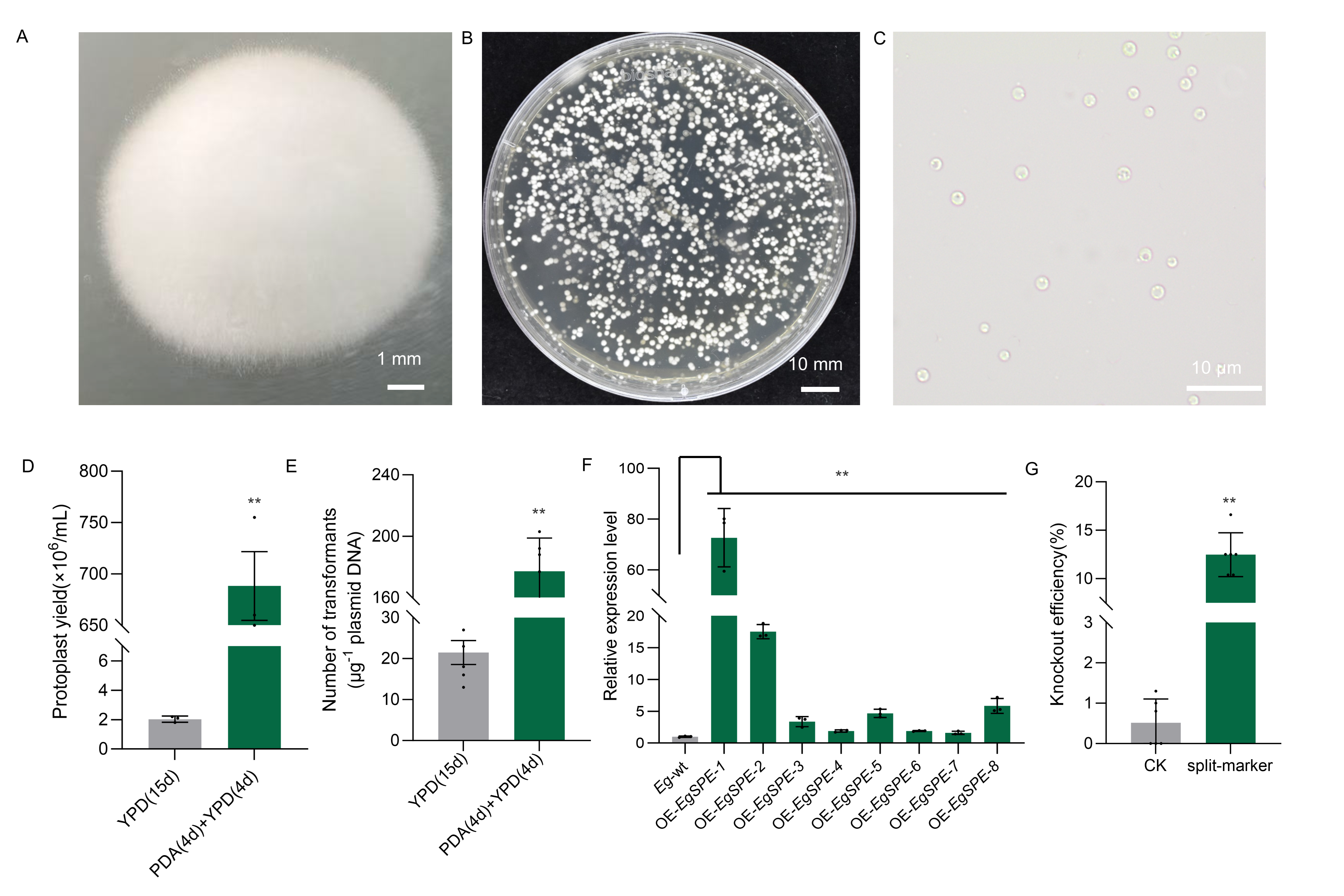


**Figure S3. Identification of genetically modified strains.**


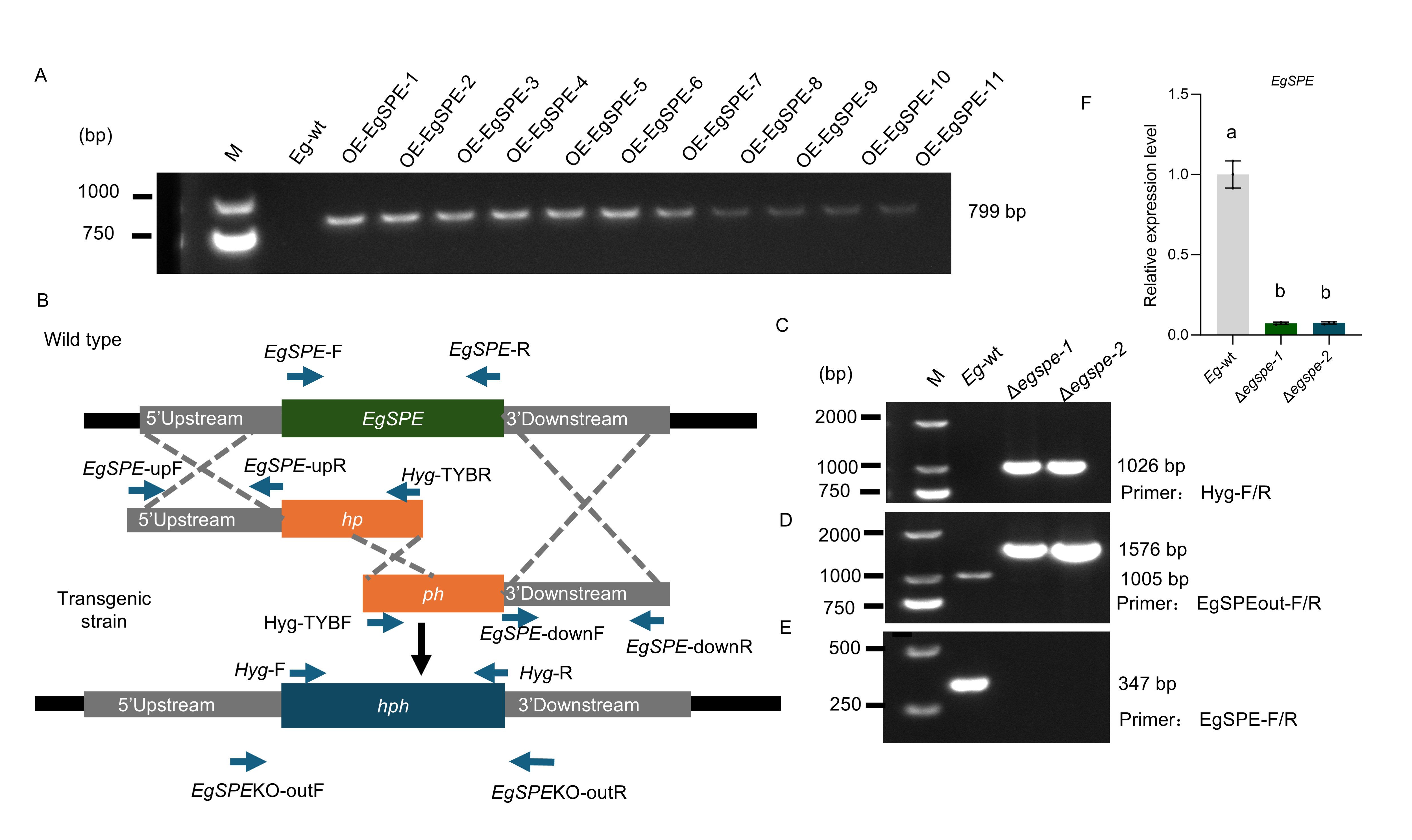


**Figure S4. Growth rate of mutants in antibiotic-free medium**


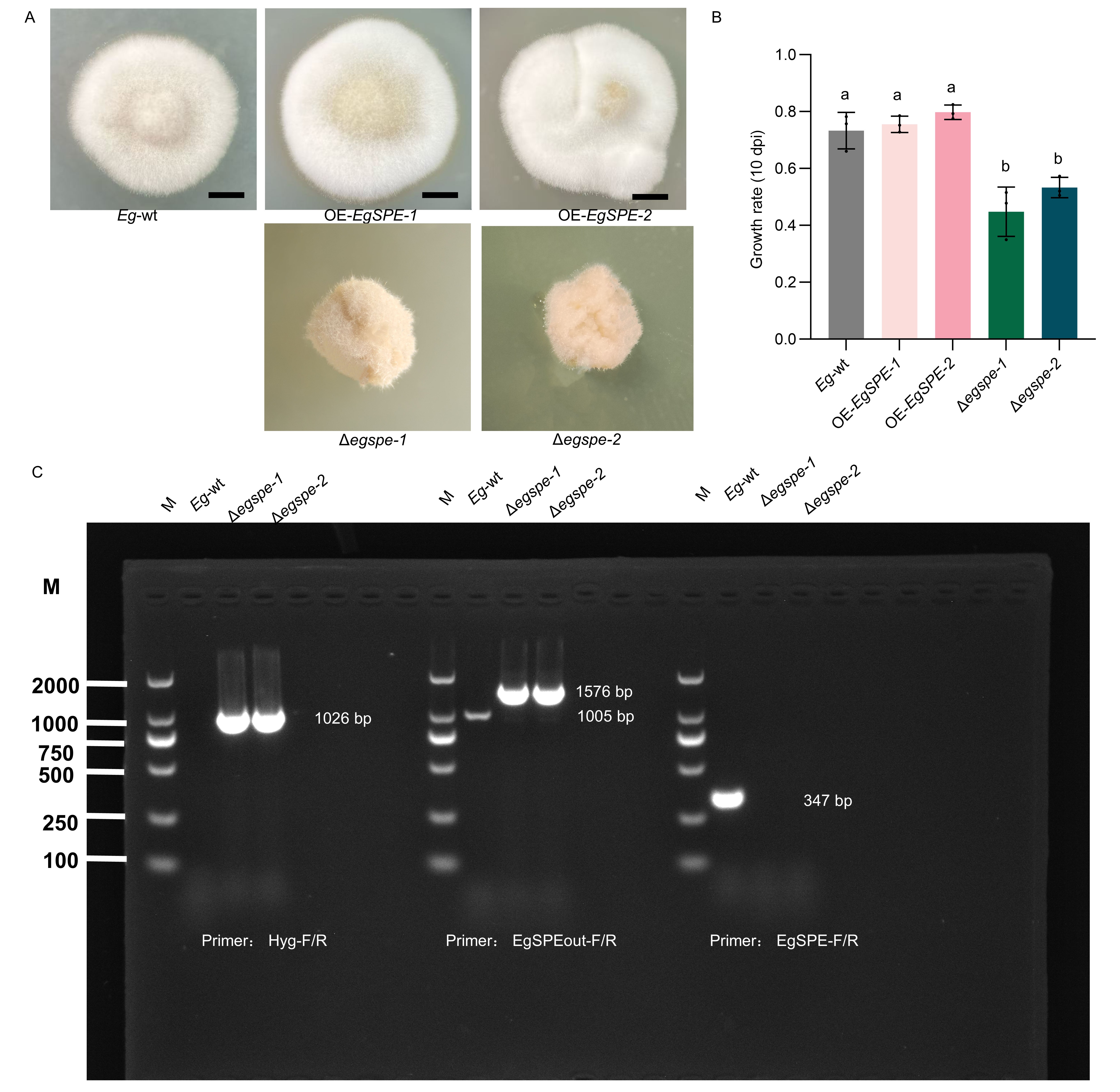


**Figure S5. Identification of successfully inoculated plants before drought treatment**


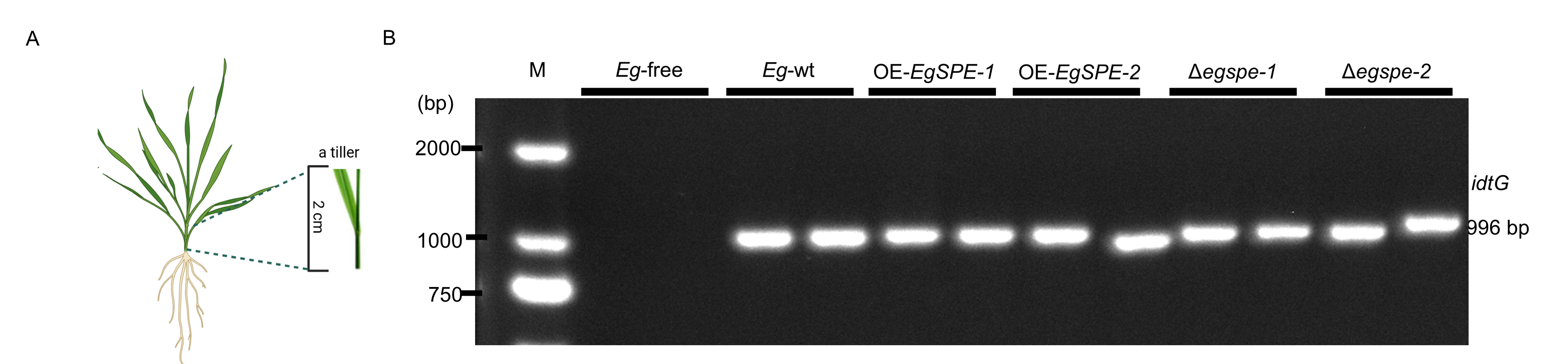

Supplement: Supplementary file 1 — Supplementary Material 1: Table S1. Conservation analysis of EgSPE in endophytic fungi. Table S2. Primer used in this study. Figure S1. Prolonged cultivation results in poor protoplast condition. (A) E. gansuensis was cultured in YPD liquid medium at 22°C with shaking at 200 rpm under dark conditions for 15 d. (B) E. gansuensis in S1A produces protoplasts with incompletely digested cell walls after 4-5 h of enzymatic hydrolysis. Figure S2. Optimized transformation system enables functional analysis of EgSPE. (A) Grow for18 d on PDA medium, 22°C, dark E. gansuensis, Scale bars = 1 mm. (B) Grow for 4 d on PDA medium covered with cellophane, 22°C, dark E. gansuensis, Scale bars = 10 mm. (C) E. gansuensis protoplasts, Scale bars = 10 μm. (D) Protoplasts yield under different treatment methods. YPD (15 d) refers to the cultivation of the YPD liquid medium for 15 d. PDA (4 d) + YPD (4 d) is culturing on a covered cellophane for 4 d, and then crushing the fungi and inoculating them onto YPD liquid medium for 4 d of cultivation. (E) Direct culture for 15 d and PDA (4 d) + YPD (4 d) transformation efficiency. (F) Expression level of different overexpressed strains. (G) Knockout efficiency of split-marker. Data in the figures are means ± SD (n=3, three biological replicates per treatment). **indicates significant differences at P≤0.05 (one-way ANOVA, Tukey post-test). Figure S3. Identification of genetically modified strains. (A) The identification of overexpression strains, using wild-type E. gansuensis as a negative control, and positive clones were identified using primers ToxA-F/pCT74jianding-HAR. (B) Schematic diagram of knockout mutant construction and the primers involved. (C), (D), (E) PCR identification of the knockout mutant. Using wild-type E. gansuensis as a negative control, three pairs of primers-Hyg-F/R, EgSPEout-F/R, and EgSPE-F/R-were used to ensure the accuracy of the identification results. (F) RT-qPCR determined the expression level of knockout mutants. Th [file 12870_2026_9133_MOESM1_ESM.docx]
